# Impact of GABA_A_ and GABA_B_ Inhibition on Cortical Dynamics and Perturbational Complexity during Synchronous and Desynchronized States

**DOI:** 10.1523/JNEUROSCI.1837-20.2021

**Published:** 2021-06-09

**Authors:** Almudena Barbero-Castillo, Pedro Mateos-Aparicio, Leonardo Dalla Porta, Alessandra Camassa, Lorena Perez-Mendez, Maria V. Sanchez-Vives

**Affiliations:** ^1^Systems Neuroscience, Institut d'Investigacions Biomèdiques August Pi i Sunyer (IDIBAPS), Barcelona, Spain 08036; ^2^Institució Catalana de Recerca i Estudis Avançats (ICREA), Barcelona, Spain 08010

**Keywords:** cerebral cortex, cortical network, slow oscillations, GABA_B_-Rs, up states, GABAergic

## Abstract

Quantitative estimations of spatiotemporal complexity of cortical activity patterns are used in the clinic as a measure of consciousness levels, but the cortical mechanisms involved are not fully understood. We used a version of the perturbational complexity index (PCI) adapted to multisite recordings from the ferret (either sex) cerebral cortex *in vitro* (sPCI) to investigate the role of GABAergic inhibition in cortical complexity. We studied two dynamical states: slow-wave activity (synchronous state) and desynchronized activity, that express low and high causal complexity respectively. Progressive blockade of GABAergic inhibition during both regimes revealed its impact on the emergent cortical activity and on sPCI. Gradual GABA_A_ receptor blockade resulted in higher synchronization, being able to drive the network from a desynchronized to a synchronous state, with a progressive decrease of complexity (sPCI). Blocking GABA_B_ receptors also resulted in a reduced sPCI, in particular when in a synchronous, slow wave state. Our findings demonstrate that physiological levels of inhibition contribute to the generation of dynamical richness and spatiotemporal complexity. However, if inhibition is diminished or enhanced, cortical complexity decreases. Using a computational model, we explored a larger parameter space in this relationship and demonstrate a link between excitatory/inhibitory balance and the complexity expressed by the cortical network.

**SIGNIFICANCE STATEMENT** The spatiotemporal complexity of the activity expressed by the cerebral cortex is a highly revealing feature of the underlying network's state. Complexity varies with physiological brain states: it is higher during awake than during sleep states. But it also informs about pathologic states: in disorders of consciousness, complexity is lower in an unresponsive wakefulness syndrome than in a minimally conscious state. What are the network parameters that modulate complexity? Here we investigate how inhibition, mediated by either GABA_A_ or GABA_A_ receptors, influences cortical complexity. And we do this departing from two extreme functional states: a highly synchronous, slow-wave state, and a desynchronized one that mimics wakefulness. We find that there is an optimal level of inhibition in which complexity is highest.

## Introduction

Brain dynamics vary according to the brain state, each one expressing in different spontaneous spatiotemporal patterns of activity, functional connectivity, responsiveness to stimuli, behavior, and cognition. These states can be physiological (e.g., awake, slow wave sleep, REM), drug-induced (e.g., anesthesia), or even different attentional states or variations within the awake state (McCormick et al., 2020). Pathologic brain states can be those associated with disorders of consciousness that can follow traumatic brain injury or stroke (Giacino et al., 2014). Different brain states are also associated with different consciousness levels. For example, slow wave sleep, deep anesthesia, and comatose states are associated with unconsciousness, while the awake state is a conscious state. Having measures that capture consciousness levels, or rather the associated brain state, is particularly relevant in the clinical realm: if an unresponsive patient would be found to be conscious, for example, it is critical to provide tools for communication. It is also important to be able to ascertain whether an anesthetized patient is unconscious as well as unresponsive. One of the parameters that has been proposed as a signature of the level of consciousness is brain complexity (Tononi and Edelman, 1998).

Network complexity represents the relationship between different components of the system, and its study is an active field across many disciplines. When applied to the brain, different measures try to capture brain complexity, either temporal, topological, or spatiotemporal complexity of spontaneous activity in either brain imaging or electrophysiology (Tononi et al., 1994; Tononi and Edelman, 1998; Bullmore and Sporns, 2009; Bettinardi et al., 2015). Another approach to quantifying cortical complexity is to induce a perturbation of the system to investigate the causal interactions that follow. The perturbational complexity index (PCI; Casali et al., 2013; Comolatti et al., 2019), in which neural activity is exogenously perturbed by means of stimulation (transcranial magnetic stimulation or electrical stimulation) has been proposed as one such measure. This method has been validated for different instances such as physiological brain states (Casali et al., 2013), anesthesia levels (Sarasso et al., 2015; Arena et al., 2020; Dasilva et al., 2021), and disorders of consciousness (Casarotto et al., 2016).

The use of PCI in humans shows that cortical bistability, which is typical of a highly synchronized state such as slow wave sleep, is a regime of low complexity. At the other extreme, the awake, conscious state, is one of high complexity. In order to explore the underlying cellular and network mechanisms behind cortical complexity, D'Andola et al. (2017) adapted the PCI measure to *in vitro* slice experiments, known as slice PCI (sPCI). They showed that when the local network switched from a slow oscillatory state to a desynchronized state induced by norepinephrine (NE) and carbachol (CCh), the bistability of Up/Down states was reduced and there was an increase in sPCI. In this way, the isolated cortical network *in vitro* was validated as a system which cannot only spontaneously generate slow oscillations (SOs; Sanchez-Vives and McCormick, 2000) and mimic other brain states (Mattia and Sanchez-Vives, 2012; Markram et al., 2015), but can also be used to investigate the cellular mechanisms of cortical complexity (D'Andola et al., 2017). In such local networks, one can specifically activate or inactivate ion/metabotropic receptors to induce changes in the spontaneous or evoked activity (sPCI) and provide insights about the underlying mechanisms controlling the transition across cortical states and potential associated changes in network complexity.

A fundamental property of cortical processing is the co-occurrence of excitation and inhibition not only in response to sensory stimulation but also during spontaneous cortical activity (Isaacson and Scanziani, 2011). To shed light on the role of inhibition in cortical complexity, here we combined electrophysiological recordings and computational simulations to investigate the relevance of fast inhibition, mediated by GABA_A_ receptors (GABA_A_-Rs), and slow inhibition, mediated by GABA_B_ receptors (GABA_B_-Rs). We first investigated the transformation of spontaneous activity patterns while gradually blocking each of these receptors. Next, we tested the ability of the cortical network to engage in complex patterns of causal interactions. To obtain a better understanding, we did this under two different dynamical patterns corresponding to synchronized and desynchronized states: (1) during a bistable state, characterized by the presence of SOs, and (2) during a desynchronized state, in which neuronal bistability was reduced. We found that blockade of both GABA_A_-Rs and GABA_B_-Rs in cortical slices reduced cortical complexity. Finally, we reproduced the experimental observations in a computational model and demonstrated dependence of complexity on the excitatory/inhibitory balance. Interestingly, physiological levels of inhibition are optimal for reaching maximum complexity, while an excess or lack of inhibition results in a decreased sPCI.

## Materials and Methods

### 

#### Slice preparation

Ferrets were treated in accordance with the European Union guidelines on protection of vertebrates used for experimentation (Directive 2010/63/EU of the European Parliament and of the council of September 22, 2010). All experiments were approved by the local ethics committee. Ferrets (4–10 months, either sex) were deeply anesthetized with isoflurane and sodium pentobarbital (40 mg/kg) before decapitation. The brain was quickly removed and placed in an ice-cold sucrose solution containing the following: 213 mm sucrose, 2.5 mm KCl, 1 mm NaH_2_PO_4_, 26 mm NaHCO_3_, 1 mm CaCl_2_, 3 mm MgSO_4_ and 10 mm glucose. Acute coronal slices (400-μm-thick) of the occipital cortex containing visual cortical areas 17, 18, and 19 from both hemispheres were cut with a Microm HM 650V vibratome (Thermo Scientific).

Slices were placed in an interface-style recording chamber (Fine Science Tools) and superfused with an equal mixture of the above-mentioned sucrose solution and artificial CSF (ACSF) containing the following: 126 mm NaCl, 2.5 mm KCl, 1 mm NaH_2_PO_4_, 26 mm NaHCO_3_, 2 mm CaCl_2_, 2 mm MgSO_4_ and 10 mm glucose. Next, slices were bathed with ACSF for 1–2 h to allow for recovery. For the slow oscillatory activity to spontaneously emerge, slices were superfused for at least 30 min before experiments with ACSF containing the following: 126 mm NaCl, 4 mm KCl, 1 mm NaH_2_PO_4_, 26 mm NaHCO_3_, 1 mm CaCl_2_, 1 mm MgSO_4_ and 10 mm glucose. All solutions were saturated with carbogen (95% O_2_/5% CO_2_) to a final pH of 7.4 at 34°C.

In addition to the SO condition, we used two more experimental conditions that were achieved as follows. (1) Cholinergic and noradrenergic agonists (“NE+CCh”), by bath-applying a mixture of modulators such as CCh (0.5 μm) and NE (50 μm). This solution blocks the generation of SO (D'Andola et al., 2017) but induces a synchronization at a higher frequency of ∼2 Hz (see below; see also Compte et al., 2008). (2) Desynchronized activity: to achieve a larger degree of asynchrony that better mimics the awake state, we used the same neuromodulators, CCh (0.5 μm) and NE (50 μm), but additionally we decreased the temperature to 32°C. Reig et al. (2010) found that decreasing the temperature to 32° induced a desynchronization of the network, which abandoned the bistable Up and Down states regime. Further, we followed Markram et al. (2015) who reduced extracellular calcium to switch from synchronous to desynchronized dynamics. To achieve this, we reduced calcium in the bath from 1–1.2 mm to 0.8–0.9 mm.

#### Electrophysiological recordings

**Figure 1.**
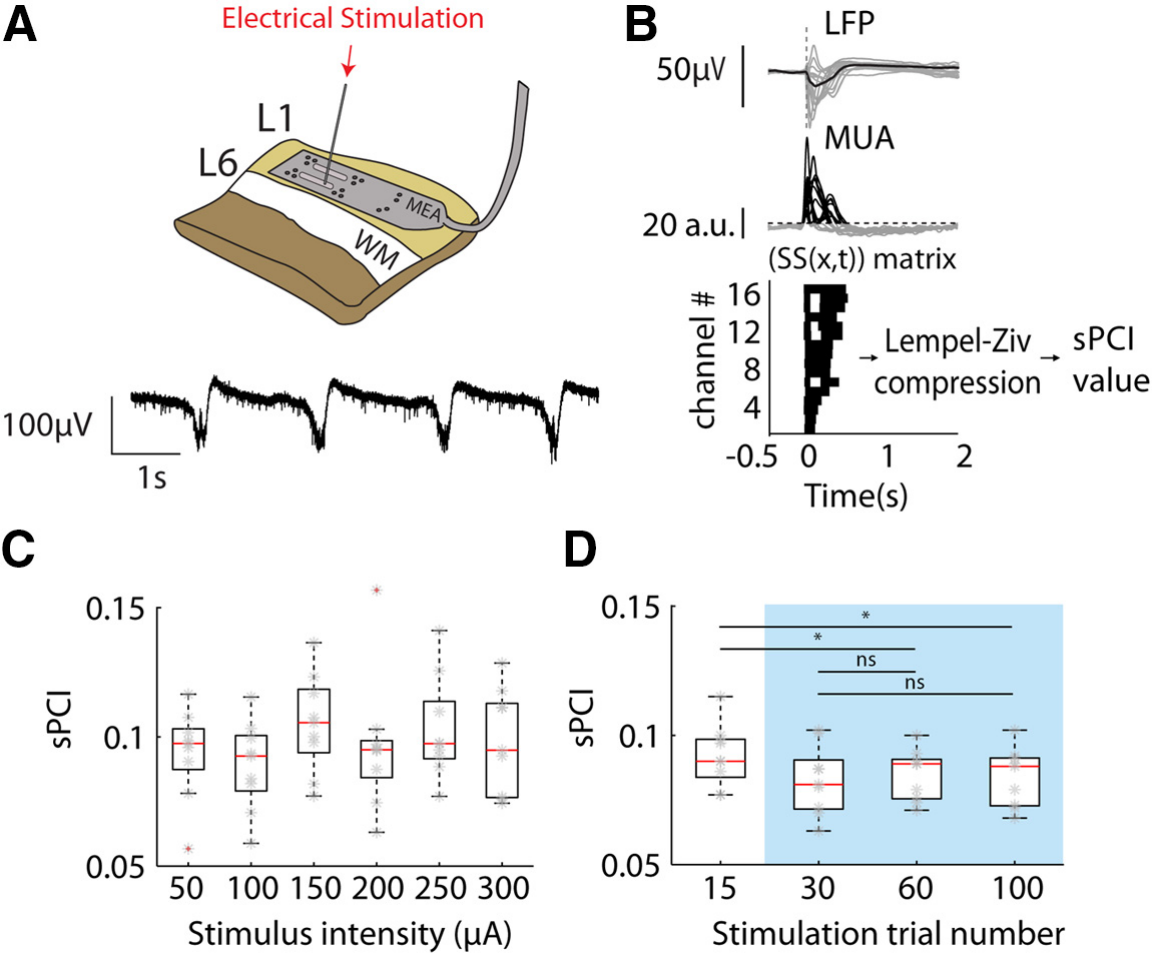
Experimental set up. ***A***, top, We recorded activity with 16-channel multielectrode array (MEA) from neocortical slices. Single pulses of electrical stimulation were applied to the infragranular layers (red arrow). Bottom, Representative LFP traces. ***B***, To calculate the network complexity (PCI in slice, sPCI), we converted the raw LFP traces (top) obtained from MEA recordings to logMUA signals (middle) and computed the binary matrices of significant activity (bottom). We then compressed the spatiotemporal binary matrices of significant sources with a Lempel–Ziv algorithm and normalized them by the source entropy to finally obtain the sPCI. ***C***, Summary plot of the sPCI calculated in control slices by applying different pulse amplitudes to the slices (*n* = 9). ***D***, Population average of the sPCI calculated in control slices with a varying number of stimulation pulses. Blue range was used during complexity protocols (*n* = 9; ns: not significant, *p < 0.05).

We recorded the extracellular local field potential (LFP) using a 16-channel SU-8-based flexible microarray (Illa et al., 2015; Capone et al., 2019). Care was taken to distribute the recording electrodes in supra and infragranular layers as shown in Figure 1*A*. Signals were amplified by 100 using a PGA16 Multichannel System (Multichannel Systems MCS GmbH-Harvard Bioscience Inc). LFPs were digitized with a Power 1401 or 1401 mkII CED interface (Cambridge Electronic Design) at a sampling rate of 5 or 10 kHz and acquired with Spike2 software (Cambridge Electronic Design).

#### Pharmacological agents

CCh and NE were obtained from Sigma-Aldrich. For the blockade of GABA_A_-Rs we used bicuculline methiodide (BMI: 0.2, 0.4, 0.6, 0.8, and 1 μm), obtained from Tocris Bioscience; and SR-95531 hydrobromide [gabazine (GBZ) 50, 100, 150, and 200 nm], obtained from Sigma-Aldrich. We also progressively blocked slow inhibition (GABA_B_-Rs) by means of CGP 55845 (CGP 100 nm, 200 nm, 500 nm, and 1 μm), obtained from Tocris Bioscience.

#### Network dynamics analysis during spontaneous activity

For every condition tested, we analyzed 300 s of spontaneous activity (no electrical stimulation pulses). We first estimated multiunit activity (MUA) from LFP recordings and detected Up and Down states as previously described (Reig et al., 2010; Ruiz-Mejias et al., 2016; D'Andola et al., 2017). Briefly, the MUA signal was calculated as the average power of the normalized spectra at a high-frequency band (200–1500 Hz), since power variations in the Fourier components at high frequencies of LFP provide a reliable estimate of the population firing rate (Mattia and Del Giudice, 2002). The MUA signal was then logarithmically scaled to balance large fluctuations of nearby spikes. We detected Up and Down states setting duration and amplitude thresholds in the log(MUA) signal. In this way, we could compute different parameters that characterize SO, such as oscillation frequency or Up and Down state durations. The firing rate was calculated as the peak log(MUA) value during Up states normalized by the log(MUA) during Down states.

The complexity of the log(MUA) time series was obtained by computing Sample Entropy (SampEn). SampEn provides an estimation of the probability that two sequences (in our case of MUA) remain similar. A lower value of SampEn indicates more self-similarity in the time series and thus, lower complexity. Briefly, SampEn is defined as the negative natural logarithm of the probability that two sequences that are similar for *m* points will remain similar for *m* + 1 points excluding self-matches (Richman and Moorman, 2000).

$$\text{SampEn}(m,r,N)=-\log\frac{A}{B},$$

where m is the length of sequences to be compared, *r* is the tolerance for accepting matches and *N* is the length of the time series. Given a time series of length *N* {x_1_, … x_N_} we define a template of length *m* = 2, X_m_(1) = {ξ, … ξ_+m-1_} and the distance function d[X_m_(1), X_m_(j)] with j ≠ i. So, *A* is the number of pairs having d[X_m+1_(1), X_m+1_(j)] < *r* and *B* is the number of pairs having d[X_m_(1), X_m_(j)] < *r*. For further details on the method, see Richman and Moorman (2000). We have used *m* = 2 and *r* = 0.25 following most SampEn analysis of biomedical signals that have used 0.1 < *r* < 0.50 (Takahashi et al., 2010; Sokunbi et al., 2013).

#### PCI for *in vitro* recordings (sPCI)

In order to estimate perturbational complexity in brain slices, we used an adaptation of the PCI used in humans (Casali et al., 2013), named sPCI (D'Andola et al., 2017). The stimulation electrode was placed in infragranular layers (Fig. 1*A*). Pulses had a duration of 0.1 ms, an intensity of 150–200 µA, and were applied every 10 s, with a random jitter from 0.5-1.5 s to avoid activity entrainment to the specific frequency of stimulation. A binary spatiotemporal distribution of significant activity was calculated in the MUA signal: we assessed the statistical differences between the network activity baseline and its response to the electrical stimulation using a bootstrap procedure as in D'Andola et al. (2017; Fig. 1*B*). The significance threshold was estimated as the one-tail (1–α) 99th percentile of the bootstrap distribution. Also, we first low-pass filtered (<10 Hz) the trial average computed on the MUA signal, and considered significant only the periods in which the activity of each channel lay above the significance threshold for >50 ms (Fig. 1*B*). The sPCI was then defined as the normalized Lempel–Ziv complexity of the binary matrix of significant evoked MUA spatiotemporal patterns (D'Andola et al., 2017; Fig. 1*B*). Furthermore, we computed the temporal evolution of the sPCI, the sPCI(t), performing the calculation of the index in temporal windows of increasing duration after the stimulation (D'Andola et al., 2017).

#### Experimental design and statistical analysis

Data are reported as mean ± SEM. Statistical significance was assessed using one-way repeated-measures ANOVA with *post hoc* Tukey's test to identify significant interactions. Exact *p* values are reported throughout the text and in graphs are represented with *p < 0.05 and **p < 0.01. Mauchly's test was used to assess the sphericity assumption in ANOVA. In datasets containing relative firing rate measurements, the Greenhouse–Geisser procedure was applied to correct for violations of sphericity. All data analyses were performed using either MATLAB (MathWorks) or Origin 8 Pro (OriginLab Corportation).

#### Computational modeling

The model consists of a two-dimensional 50 × 50 squared network of pyramidal cells (80%) and interneurons (20%), randomly distributed and interconnected through biologically plausible synaptic dynamics. Each cell is sparsely and locally connected to its neighbors within a square of size *L* × *L* centered around it, where *L*_pyramidal_ = 7 and *L*_interneuron_ = 5. The fraction of synaptic connections (outgoing synapses) is set at 50% of the total number of neurons within the local range for pyramidal cells and 90% for interneurons, thus imposing local connections for interneurons and more sparse connections for pyramidal cells. The network structure is similar to that used in previous studies of oscillatory neuronal networks (Bazhenov et al., 2008; Poil et al., 2012; Dalla Porta and Copelli, 2019). The neuron model and its ion channel dynamics are borrowed with maximal conductance adjustment from Compte et al. (2003; this model is currently available in NEST by Maksimov et al., 2016). Our detailed ionic channel dynamics and parameters are given in Extended Data Figure 7-1. Additionally, the model accounts for the potassium leak current *I*_KL_, which is modulated by acetylcholine (ACh) and NE (Bazhenov et al., 2002; Li et al., 2017) and by GABA_B_ inhibitory synapses (see Extended Data Fig. 7-1). Briefly, the pyramidal cells, consisting in a somatic and a dendritic compartment, are modeled as:

(1) $$C_{m}A_{s}\frac{dV_{s}}{dt}=-A_{s}(I_{L}+I_{KL}+I_{Na}+I_{K}+I_{A}+I_{KS}+I_{KNa})-I_{syn,i}-g_{sd}(V_{s}-V_{d}),$$

and,

(2) $$C_{m}A_{d}\frac{dV_{d}}{dt}=-A_{d}(I_{L}+I_{ca}+I_{Kca}+I_{NaP}+I_{AR})-I_{syn,E}-g_{sd}(V_{d}-V_{s}),$$

where *V*_s_ and *V*_d_ represent the soma and dendrite voltage, respectively. *C*_m_ is the specific membrane capacitance, *g*_sd_ (1.75 ± 0.1 µS) is the conductance of the coupling between soma and dendrite, and *A*_s,d_ are the membrane areas of soma and dendrite, respectively. *I*_syn,i_ and *I*_syn,E_ accounts for inhibitory and excitatory synaptic currents, respectively. As in Compte et al. (2003), in our simulations, all excitatory synapses target the dendritic compartment and all inhibitory synapses are localized on the somatic compartment of postsynaptic pyramidal neurons.

10.1523/JNEUROSCI.1837-20.2021.f7-1 Figure 7-1 Computational network model. Download Figure 7-1, DOCX file.

The soma includes the following channels and respective maximal conductances (*g*): leakage current (*I*_L_, *g*_L_ = 0.0667 ± 0.0067 mS/cm^2^), potassium leakage current (*I*_KL_, *g*_KL_ = 1.86 mS/cm^2^), sodium current (*I*_Na_, *g*_Na_ = 50 mS/cm^2^), potassium current (*I*_K_, *g*_K_ = 10.5 mS/cm^2^), A-type K^+^ current (*I*_A_, *g*_A_ = 0.95 mS/cm^2^), non-inactivating slow K^+^ current (*I*_KS_, *g*_KS_ = 0.5472 mS/cm^2^) and the Na^+^-dependent K^+^ current (*I*_KNa_, *g*_KNa_ = 0.65835 mS/cm^2^). The dendrite includes: leakage current (*I*_L_, *g*_L_ = 0.0667 ± 0.0067 mS/cm^2^), high-threshold Ca^2+^ channel (*I*_Ca_, *g*_Ca_ = 0.43 mS/cm^2^), Ca^2+^-dependent K^+^ current (*I*_KCa_, *g*_KCa_ = 0.5415 mS/cm^2^), persistent Na^+^ channel (*I*_NaP_, *g*_NaP_ = 0.05145 mS/cm^2^) and the anomalous rectifier K^+^ channel (*I*_AR_, *g*_AR_ = 0.0257 mS/cm^2^). *I*_syn,I_ and *I*_syn,E_ are the inhibitory and excitatory synaptic currents, respectively. The interneurons, consisting in only one compartment, are simply modeled as:

(3) $$C_{m}A_{I}\frac{dv_{s}}{dt}=-A_{i}(I_{L}+I_{Na}+I_{K})-I_{syn},$$

where *A_i_* is the total membrane area and *I*_syn_ accounts for both the inhibitory and excitatory synaptic currents. All the details of the implementation of these currents are described by Compte et al. (2003), except for *I*_KL_ and GABA_B_, which are described below. The GABA_B_ current (*I*_GABAB_) is modeled as (Destexhe et al., 1996; Liu et al., 2019), where *r* and *s* represent the GABA_B_-R and the gating variable, respectively. The transmitter concentration *T* is modeled as a square pulse of 0.5 mm during 3 ms. *I*_KL_ is modeled as in Li et al. (2017) where *I*_KL_ = *g*_KL_(*V – V*_K_), with *V*_K_ = –100 mV (potassium reversal potential). The synaptic maximal conductances (*g_x_^jk^*, where *x* stands for AMPA, NDMA, GABA_A_, and GABA_B_, and *j* and *k* stand for the presynaptic and postsynaptic neuron, respectively) are set for values detailed in Extended Data Fig. 7-1. Excitatory synapses are mediated by AMPA and NMDA, while inhibitory synapses by GABA_A_ and GABA_B_. Additionally, all neurons receive a heterogeneous Poisson train of excitatory, AMPA and NMDA, presynaptic potentials with a rate of 0.5 kHz (Dayan and Abbott, 2001). The Poisson synaptic inputs are modeled as excitatory AMPA and NMDA currents where the probability of a spike at one time-step is given by: 1 – exp (–R * *dt*), where *dt* is the time step of simulation, and R the Poisson rate. All the channels and synapse kinetics as well as parameters are described in detail in Extended Data Fig. 7-1.

In order to simulate the experimental effects of GABA_A,B_-Rs blockade, we progressively reduced the GABA_A,B_ conductance (which will be referred to as simple concentration) in inhibitory synapses to both neurons, pyramidal and interneurons, from 5% to 90%. We proceeded in the same way to progressively increase the GABA_A_ channel conductance in Figure 8*B*, increasing it from 5% to 90%.

For the model network to switch from synchronized to desynchronized state, we completely blocked the potassium leak current, mimicking an action of ACh and NE (McCormick, 1992). This strategy has been used in cortical (Bazhenov et al., 2002) and thalamic models (Li et al., 2017) to induce the transition from synchronized to desynchronized states. For the stimulation procedure, we depolarized all the neurons by a brief (40 ms) external stimulation current of 0.5 nA with an interval of stimulation of 5 ± 1 s (mean ± SD given).

The simulated population membrane potential (sLFP) was computed as the sum of the absolute values of the excitatory and inhibitory synaptic currents acting on the excitatory neurons (Sancristóbal et al., 2016). We virtually created 20 electrodes in the model, arranged as a 5 × 4 matrix. Each electrode covered an area of 49 neurons and were horizontally and vertically spaced by a distance of 10 neurons, thus ensuring no overlapping between electrodes. The neurons on the border were not considered. The model was implemented in a C code and simulated using a fourth-order Runge–Kutta method with a time step of 0.06 ms during 210 s. To compute the mean response of the membrane potential, we averaged over 10 realizations of the external noise, network connectivity and neuron parameters.

## Results

### Synchronous versus desynchronized states in the cortical network: spontaneous activity and perturbational complexity

**Figure 2.**
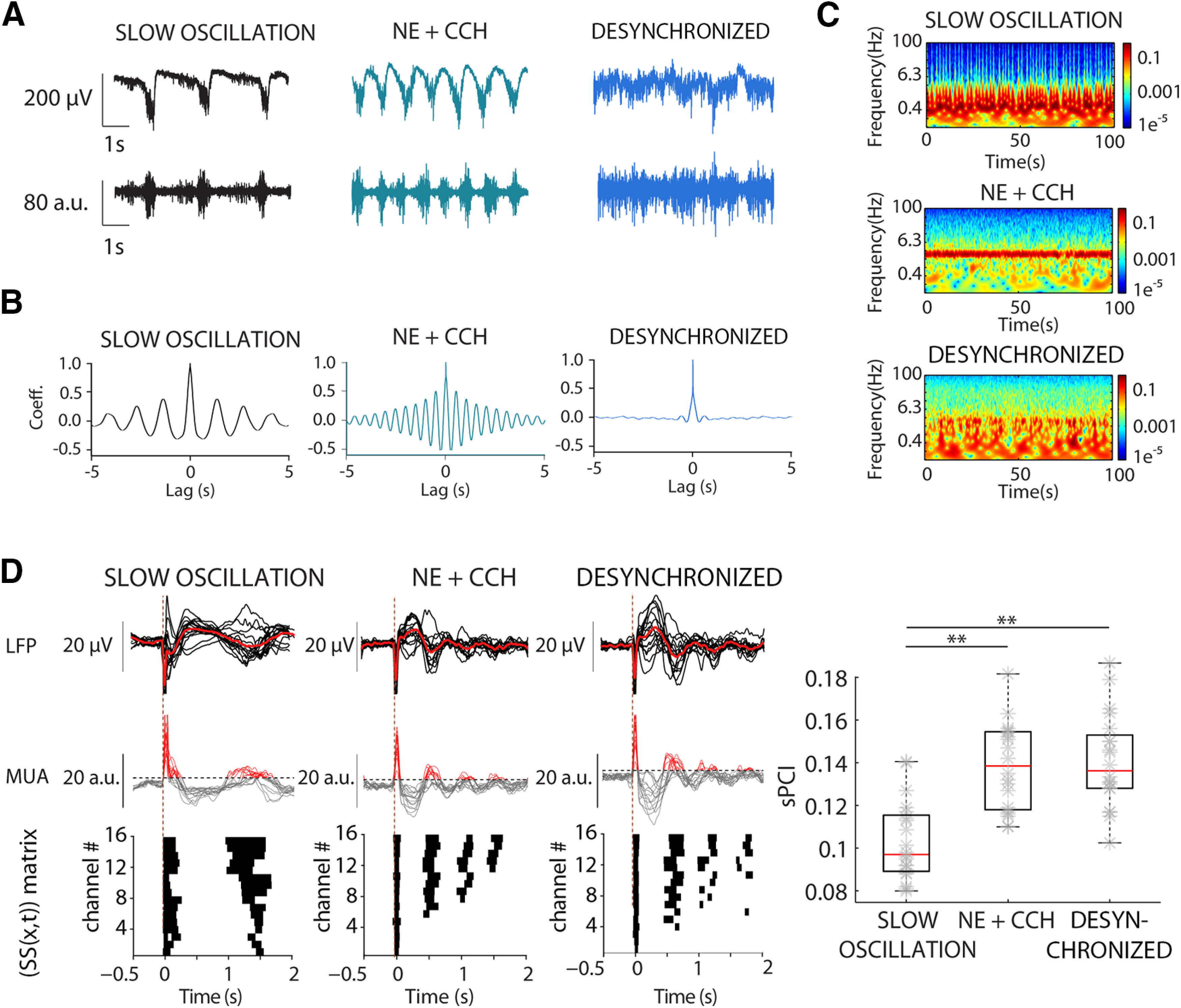
Network complexity increased from spontaneous synchronous SO to desynchronized activity. ***A***, Raw LFP (top) and MUA (bottom) recordings of 5 s of spontaneous SO (left), NE+CCh (middle), and desynchronized activity (right). ***B***, Representative autocorrelograms showing the lack of slow oscillatory activity during desynchronized activity (right). ***C***, Spectrograms of spontaneous activity from LFP recordings shown in ***A***. ***D***, left, Averaged LFP (top) and MUA (bottom) responses to electrical stimulation during spontaneous SO (left), NE+CCh (middle), and desynchronized activity (right). Binary matrices of significant sources of activity [SS(*x*,*t*)] following electrical stimulation delivered to neocortical slices (bottom). Right, Population sPCI (*n* = 20) measured during control SO, NE+CCh, and desynchronized activity (**p < 0.01).

*In vitro* extracellular 16-channel LFPs were recorded from ferret primary visual cortex (V1) coronal slices (*n* = 58) during two different regimes of spontaneous activity: (1) synchronous activity consisting in spontaneous Up and Down states organized in SOs (Figs. 1*A*, 2*A–C*); and (2) desynchronized activity (Fig. 2*A–C*).

In our experimental paradigm, cortical slices displayed spontaneous SO similar to the ones occurring *in vivo* during slow wave sleep (Sanchez-Vives and McCormick, 2000). Oscillatory frequencies in the different slices ranged from 0.2 to 0.92 Hz (mean 0.47 ± 0.02 Hz, *n* = 58). While the synchronous slow oscillatory state replicates the dynamics of slow wave sleep, desynchronized activity in the slice can mimic that of awake states. In cortical slices, desynchronized states can be mimicked by adding neurotransmitters, through bath application of NE and ACh, present in awake states (McCormick, 1992; Brumberg et al., 2000; Jones, 2005; D'Andola et al., 2017). Even when cholinergic and noradrenergic agonists are reported to block SO through the blockade of afterhyperpolarizations (Steriade et al., 1993b), we still observe synchronization at a frequency higher than spontaneous SO, at ∼2.4 Hz (see Fig. 2*A*,*B*). In order to obtain a more desynchronized activity, we used additional strategies based on previous studies, recording at 32°C (Reig et al., 2010) and lowering calcium in the bath from 1–1.2 mm to 0.8–0.9 mm (Markram et al., 2015) to enhance excitability (see Materials and Methods; Fig. 2*A–C*). Using a measure of complexity of time series called SampEn (see Materials and Methods), we quantified the level of regularity of the signal during three different types of spontaneous activity or conditions that we will used throughout the study: (1) SO; (2) NE+CCh to refer to the presence of cholinergic (CCh) and noradrenergic (NE) agonists in the bath; and (3) desynchronized activity, mimicking awake states (for details, see Materials and Methods). The mean SampEn was SO: 0.751 ± 0.027; NE+CCh: 0.868 ± 0.033; desynchronized: 0.906 ± 0.035; *F*_(2,38)_ = 13.087, *p* = 4.742 × 10^−5^, *n* = 20. In particular, SampEn measured during NE+CCh and desynchronized activity was significantly higher than during SO (NE+CCh: *t*_(38)_ = 5.218, *p* = 0.002; desynchronized: *t*_(38)_ = 6.95, *p* = 5.07 × 10^−5^, *n* = 20), as we would expect given that SO are more synchronized states.

In order to quantify the complexity of network responses to single-pulse electrical stimulation, we used an adapted version of the PCI (Casali et al., 2013) for slice recordings (sPCI; Fig. 1*B*; see Materials and Methods; D'Andola et al., 2017). During ongoing SO, electrical stimulation evoked a response followed by a sudden decrease in activity, Down state (Fig. 1*B*) or what, in humans, has been referred to as “off-periods” (Rosanova et al., 2018), resembling reported findings for LFP recordings in humans (Pigorini et al., 2015; Rosanova et al., 2018). According to the sPCI algorithm by (D'Andola et al., 2017) and to quantify the spatiotemporal patterns of response to electrical stimulation, we converted the raw LFP traces obtained from multielectrode array recordings to firing rate signals (specifically to logMUA, see Materials and Methods; Fig. 1*B*) and computed the binary matrices of significant activity (Fig. 1*B*; for details, see Materials and Methods). We then compressed the spatiotemporal binary matrices of significant sources with a Lempel–Ziv algorithm and normalized them by the source entropy to finally obtain the sPCI. Under synchronous, slow oscillatory activity, the sPCI was 0.1 ± 0.002 (range 0.07–0.14, *n* = 58), similar to what has been previously reported (D'Andola et al., 2017; Dasilva et al., 2021).

We tested the reliability of the sPCI in a subset of slices by (1) applying different stimulation intensities (Fig. 1*C*); and (2) increasing the number of stimuli repetitions (Fig. 1*D*). The population sPCI did not vary significantly when different stimulation intensities were applied within the range of 50–300 µA (*n* = 9; Fig. 1*C*). However, the sPCI was slightly dependent on the number of stimulation trials. For 15 repetitions this was 0.092 ± 0.004, 0.083 ± 0.004 for 30 repetitions, 0.085 ± 0.003 for 60 repetitions, and 0.084 ± 0.004 for 100 repetitions (*F*_(3,24)_ = 4.44, *p* = 0.012, *n* = 9; Fig. 1*D*). The sPCI significantly decreased above 15 repetitions but remained stable for 30, 60, and 100 repetitions (15 vs 30 rep.: *t*_(24)_ = 2.66, *p* = 0.261; 15 vs 60 rep.: *t*_(24)_ = 4.34, *p* = 0.025; 15 vs 100 rep.: *t*_(24)_ = 4.57, *p* = 0.017; 30 vs 60 rep.: *t*_(24)_ = 1.67, *p* = 0.644; 30 vs 100 rep.: *t*_(24)_ = 1.91, *p* = 0.541; 60 vs 100 rep.: *t*_(24)_ = 0.24, *p* = 0.998; n = 9; Fig. 1*D*). Therefore, in the rest of the study we consistently calculated PCI with 40 as the number of stimuli.

### sPCI in different dynamic regimes of cortical activity

We next calculated sPCI in the three described conditions: (1) SO; (2) NE+CCh; and (3) desynchronized state (Fig. 2). As said above, following the bath application of NE+CCh, the regime of Up/Down states was transformed (Fig. 2*A–C*), the network went on to generate a higher frequency (∼2.4 Hz) of smaller amplitude, with an increased SampEn. The sPCI following the electrical stimulation revealed a significant increase of the sPCI with respect to that in SO (Fig. 2*D*), similar to what was reported previously (D'Andola et al., 2017). In the desynchronized state, the mean sPCI was also significantly larger than that in SO, but not higher than in NE+CCh, despite being more desynchronized (SO: 0.103 ± 0.004; desynchronized: 0.141 ± 0.005, *t*_(38)_ = 8.599, *p* = 1.301 × 10^−6^, *n* = 20; Fig. 2*D*). When cortical complexity was calculated by means of sPCI, there was a significant increase in complexity following the blockade of network Up/Down state bistability, which is a highly synchronous state. However, the more subtle change in dynamics taking place between NE+CCh and desynchronized conditions did not convey a complexity increase as detected by sPCI. From this point on in the study, we used two departing points or baselines that we compared: (1) the slow oscillatory, synchronous state; and (2) the desynchronized state. These two extremes of the dynamics mimic awake versus slow wave sleep, or awake versus deep anesthesia, respectively.

### Role of GABA_A_-Rs in the modulation of cortical dynamics and complexity: blocking of GABA_A_-Rs in the desynchronized state

**Figure 3.**
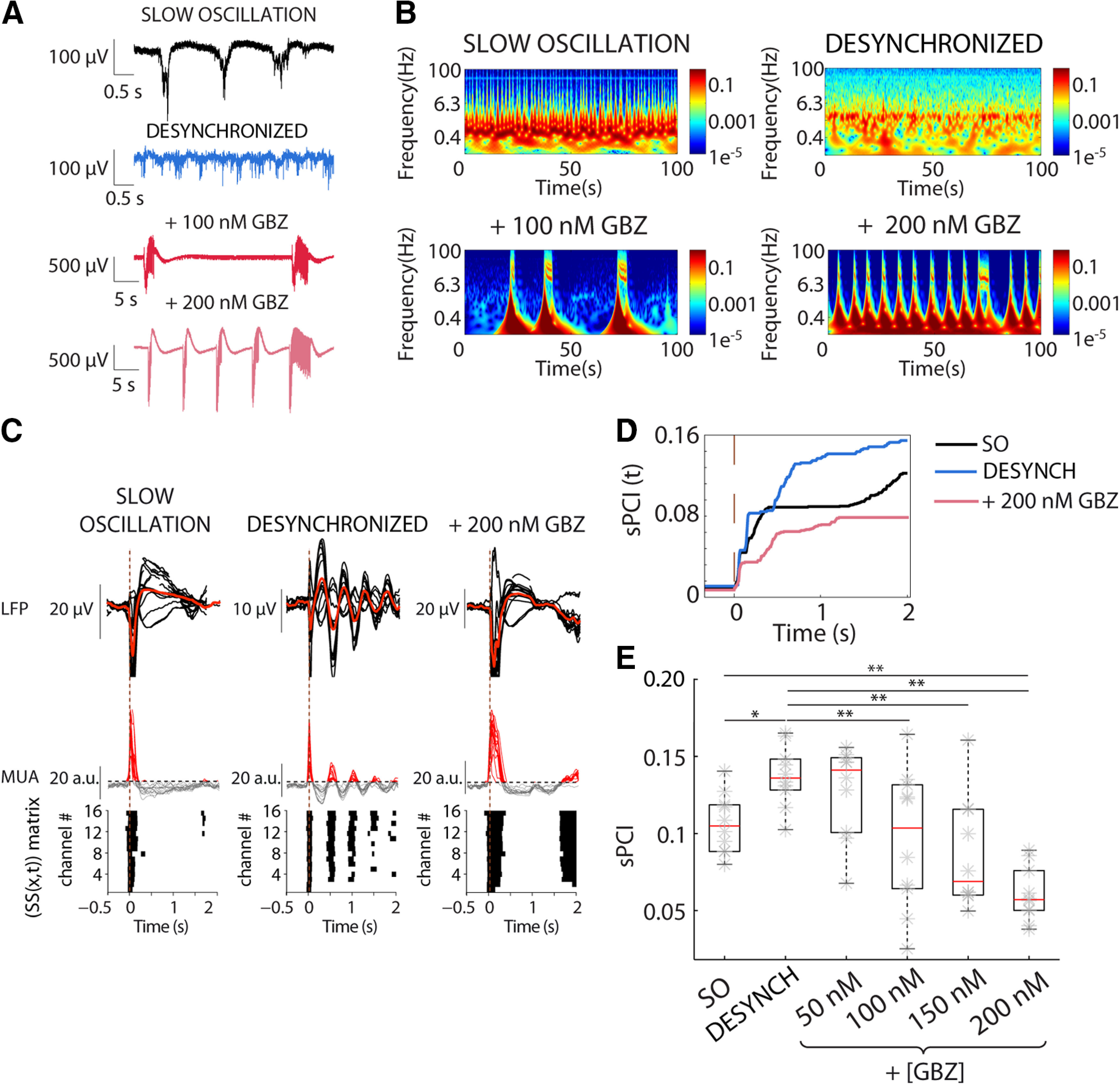
Progressive blockade of GABA_A_-Rs reduces sPCI during desynchronized activity recovering bistability. ***A***, Raw LFP recordings of spontaneous activity in neocortical slices, during SO, desynchronized activity and blockade of GABA_A_-Rs by bath application of increasing concentrations of GBZ demonstrated progressively shifted toward preepileptiform dynamics. ***B***, Spectrograms of spontaneous activity from LFP recordings shown in ***A***. ***C***, Averaged LFP (top) and MUA (middle) to electrical stimulation during distinct regimes of activity. Binary matrices of significant sources of activity [SS(*x*,*t*)] following electrical stimulation delivered to neocortical slices (bottom). ***D***, Time evolution of sPCI in the three experimental conditions ***E***, Population sPCI (*n* = 10) demonstrated that presence of large Down-states breaks the causal interactions and decreased the complexity of the responses (*p < 0.05, **p < 0.01).

To investigate the GABAergic role in cortical complexity we explored how a progressive blockade of inhibition affected sPCI while departing from two different dynamic states, either (1) the desynchronized state or (2) the synchronous, slow oscillatory state. We first induced the desynchronized state (Fig. 3*A*,*B*) as described in Materials and Methods, and next we blocked fast inhibition by application of the selective GABA_A_-R blocker GBZ (Fig. 3; 50–200 nm). When GABA_A_-Rs were blocked, desynchronized dynamics progressively shifted toward pre-epileptiform dynamics (Fig. 3*A*,*B*) as described in Sanchez-Vives et al. (2010; Fig. 6*D*). We illustrate how this effect was expressed in the raw traces (Fig. 3*A*) and in the spectrogram of the activity (Fig. 3*B*). Such modification of spontaneous dynamics was also reflected in the spatiotemporal pattern of responses to perturbation (Fig. 3*C*), that were used for the calculation of sPCI (Fig. 3*C–E*). As shown above, from slow oscillatory regime to desynchronized regime, there was an increase in sPCI. However, following the maximum sPCI reached in the desynchronized state, the progressive blockade of GABA_A_-Rs resulted in a progressive decline of sPCI (*n* = 10; Fig. 3*D*,*E*). Interestingly, whereas the sPCI was significantly reduced compared with the desynchronized state for concentrations above 50 nm GBZ (GBZ 100 nm: *t*_(45)_ = 5.56, *p* = 0.003; GBZ 150 nm: *t*_(45)_ = 6.979, *p* = 1.588 × 10^−4^; GBZ 200 nm: *t*_(45)_ = 10.485, *p* = 4.128 × 10^−8^; *n* = 10; Fig. 3*E*), it was only at the highest concentration of GBZ that the sPCI significantly decayed below control levels (*t*_(45)_ = 6.294, *p* = 7.525 × 10^−4^, *n* = 10; Fig. 3*E*). The trend shown in Figure 3*E* was in agreement with the temporal evolution of the sPCI (Fig. 3*D*) that showed a faster increase in the desynchronized state with respect to the other two conditions in which the increase of sPCI reached a plateau around 0.6 s after the stimulation. In summary, these results indicate that blockade of GABA_A_-Rs in the desynchronized state decreases perturbational complexity in cortical slices, or conversely, physiological GABA_A_-mediated inhibition contributes to cortical complexity during desynchronized dynamics. Further, highly synchronous epileptiform discharges (in 200 nm GBZ) display decreased complexity. A decreased information content and complexity in synchronous, epileptic discharges has also been described in both animal models and humans (Lehnertz and Elger, 1995; Artinian et al., 2011; Trevelyan et al., 2013).

### Role of GABA_A_-Rs in the modulation of cortical dynamics and complexity: blocking of GABA_A_-Rs in the slow oscillatory state

We next investigated the effect of GABA_A_-R blockade on complexity but departing from SO. We bath-applied increasing concentrations of BMI (0.2, 0.4, 0.6, 0.8, and 1 μm) and recorded network responses to electrical stimulation. Raising BMI concentrations induced a gradual shortening of evoked Up states and augmented Up-state amplitude, as previously described (Sanchez-Vives et al., 2010). Such an increase in Up-state amplitude corresponded to a linear increase in the firing rate during Up states with the removal of inhibition because of an enhanced excitatory reverberation. The increase in firing rate was significant for all conditions compared with SO (*n* = 9; BMI 0.2 μm: *t*_(40)_ = 5.25, *p* = 0.008; BMI 0.4 μm: *t*_(40)_ = 9.96, *p* = 2.87 × 10^−7^; BMI 0.6 μm: *t*_(40)_ = 14.98, *p* = 2.21 × 10^−10^; BMI 0.8 μm: *t*_(40)_ = 18.15, *p* < 0.001; BMI 1 μm: *t*_(40)_ = 19.83, *p* < 0.001). Up states of larger amplitude resulted in binary matrices with shorter significant periods of activity. In particular, bath-application of BMI reduced the sPCI with respect to SO (*n* = 9). *Post hoc* analysis revealed that the trend of decay of sPCI with the removal of fast inhibition became significantly reduced at 1 μm BMI with respect to SO (*t*_(40)_ = 4.93, *p* = 0.014, *n* = 9; data not shown).

**Figure 4.**
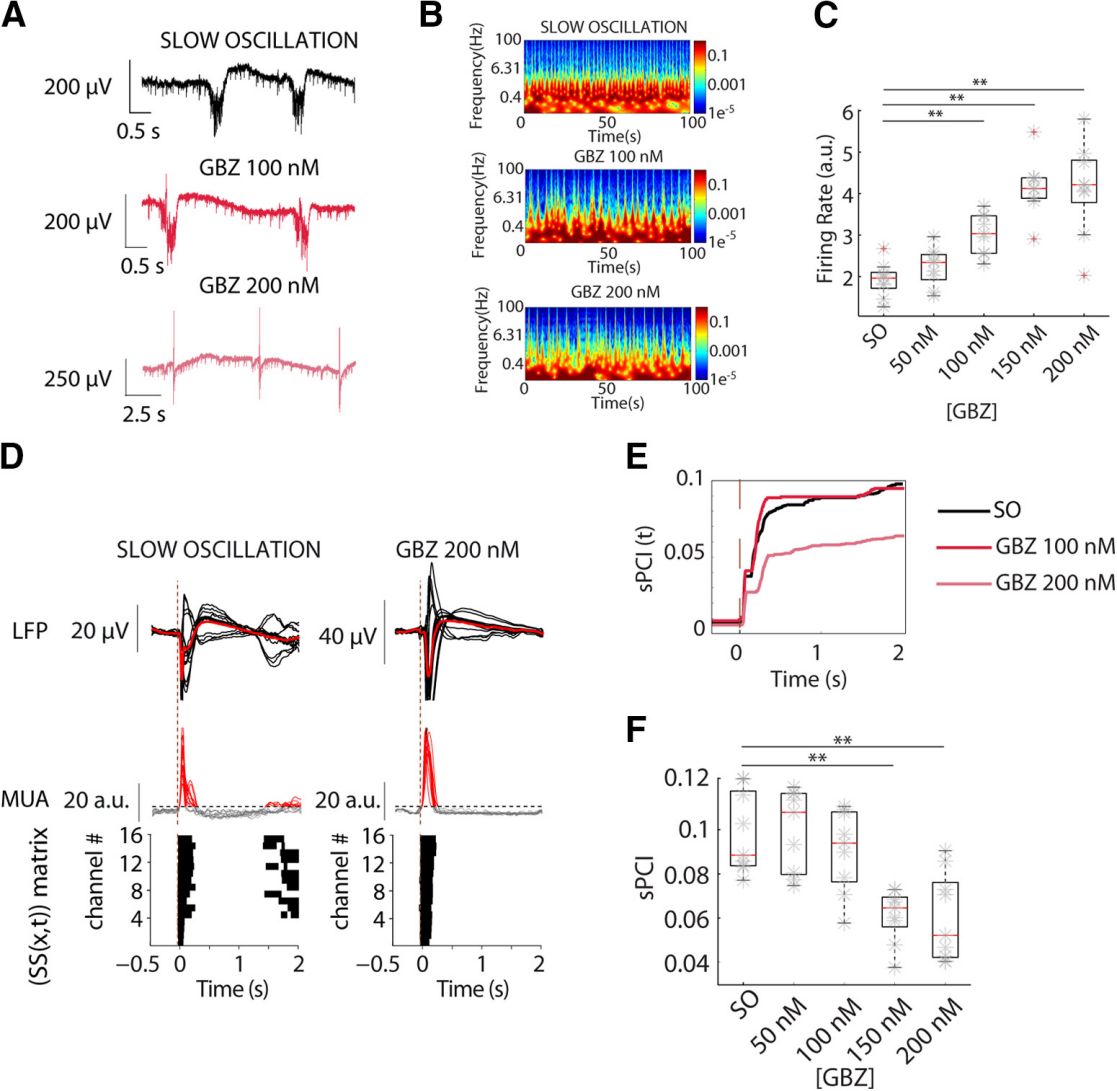
Progressive blockade of GABA_A_-Rs reduces sPCI during SWA. ***A***, Raw LFP recordings of spontaneous activity in neocortical slices, control SOs and blockade of GABA_A_-Rs by bath application of increasing concentrations of GBZ induced shortening of evoked Up states. ***B***, Spectrograms of the spontaneous activity shown in ***A***. ***C***, Population FRs for increasing GBZ concentrations. ***D***, Averaged LFP (top) and MUA (middle) responses to electrical stimulation during distinct regimes of activity. Binary matrices of significant sources of activity [SS(*x,t*)] following electrical stimulation delivered to neocortical slices (bottom). ***E***, Time evolution of sPCI in the three experimental conditions. ***F***, Population sPCI (*n* = 9) demonstrated that presence of large Down states breaks the causal interactions and correlates with low complexity states (**p < 0.01).

BMI is known to block additional targets such as the small conductance calcium-activated potassium (SK) channels (Khawaled et al., 1999). To avoid potential confounding effects in our experiments, we next bath-applied increasing concentrations of GBZ, a specific blocker of GABA_A_ that lacks the effect on SK channels. Blockade of GABA_A_-Rs by increasing concentrations of GBZ also induced shortening of evoked Up states (Fig. 4*A*,*B*), as previously shown (Sanchez-Vives et al., 2010). As occurred after application of BMI, the firing rate during Up states also gradually increased, eventually leading to epileptiform discharges in some cases (*n* = 9; Fig. 4*B*). Although the firing rate increase showed a clear trend for all GBZ concentrations, this increase was significant above 50 nm (GBZ 50 nm: *t*_(32)_ = 1.43, *p* = 0.85; GBZ 100 nm: *t*_(32)_ = 5.07, *p* = 0.009; GBZ 150 nm: *t*_(32)_ = 10.17, *p* = 3.43 × 10^−7^; GBZ 200 nm: *t*_(32)_ = 10.01, *p* = 4.7 × 10^−7^; *n* = 9; Fig. 4*C*).

The change in sPCI induced by the removal of fast inhibition with GBZ was largely similar to the one induced by BMI. Overall, the sPCI significantly decreased with increasing GBZ concentrations (*n* = 9; Fig. 4*D–F*). In particular, the sPCI reduction with respect to the SO condition was significant above 100 nm GBZ (GBZ 150 nm: *t*_(32)_ = 7.04, *p* = 1.94 × 10^−4^; GBZ 200 nm: *t*_(32)_ = 7.22, *p* = 1.36 × 10^−4^; *n* = 9).

Thus, these results indicate that removal of fast inhibition reduces perturbational complexity in cortical slices. Enhanced excitability during Up states because of excitatory recurrency in cortical circuits induced stereotypical responses to stimulation that resulted in lower sPCI values. Balanced GABA_A_-R-mediated inhibition in cortical activity provides richness in the emergent patterns, contributing to the complexity of causal interactions. Later, in our computer model, we explored the limits of the relationship between inhibition and complexity, in a range that is unattainable experimentally.

### Role of GABA_B_-Rs in the modulation of cortical dynamics and complexity: blocking of GABA_B_-Rs in the desynchronized state

**Figure 5.**
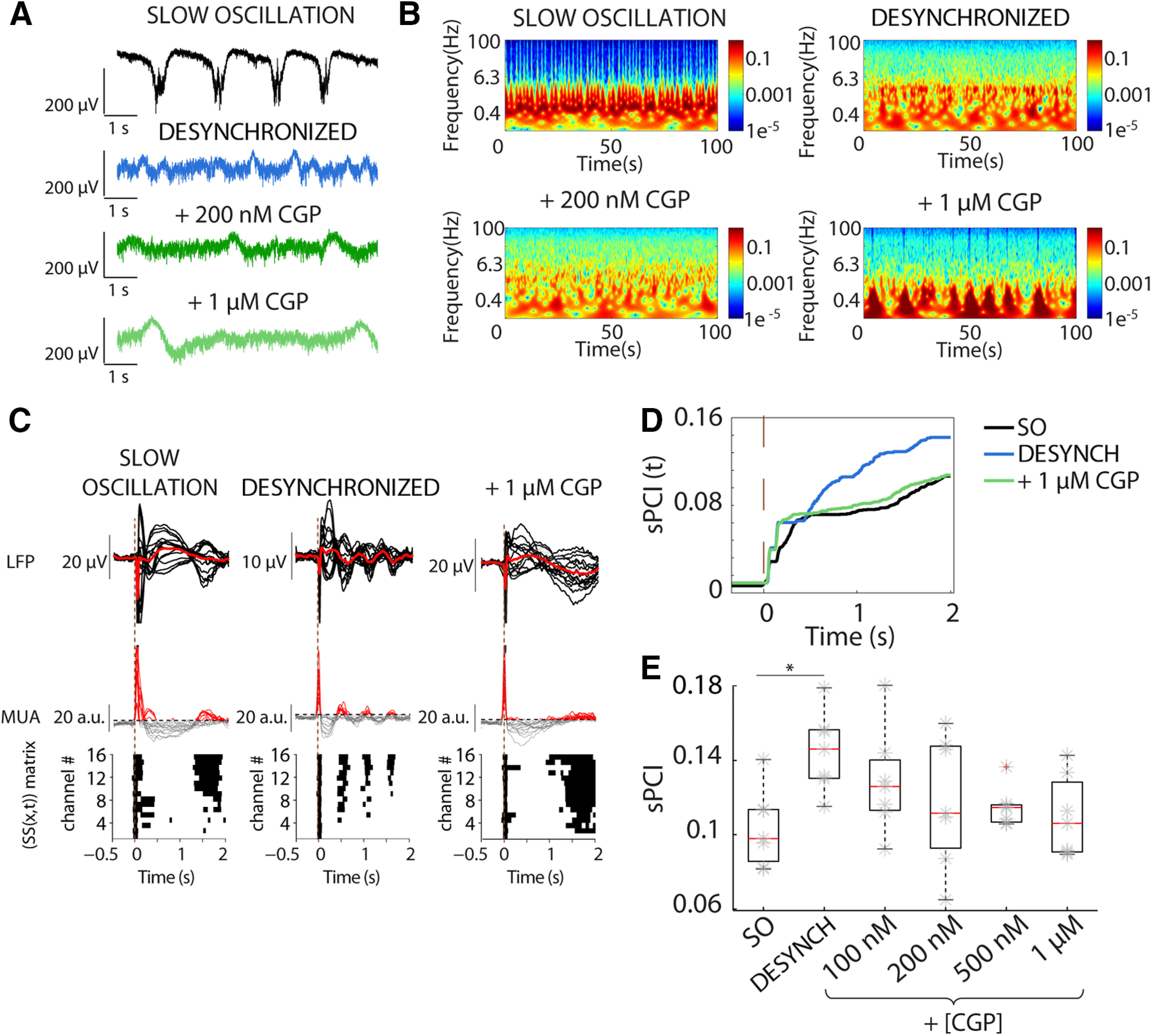
Progressive blockade of GABA_B_-Rs did not significantly reduce the sPCI during desynchronized activity. ***A***, Raw LFP recordings of spontaneous activity in neocortical slices SOs, desynchronized activity and blockade of GABA_B_-Rs by bath application of increasing concentrations of CGP, which enhanced the synchronization in the network. ***B***, Spectrograms of spontaneous activity from LFP recordings shown in ***A***. ***C***, Averaged LFP (top) and MUA (middle) to electrical stimulation during distinct regimes of activity. Binary matrices of significant sources of activity [SS(*x*,*t*)] following electrical stimulation delivered to neocortical slices (bottom). ***D***, Time evolution of sPCI in the three experimental conditions. ***E***, Population sPCI (*n* = 7) demonstrated that higher synchronization reduces cortical complexity (*p < 0.05).

Next, we followed a similar approach to investigate the effects of progressive GABA_B_-R blockade during desynchronized activity in cortical slices (Fig. 5). Departing from slow oscillatory spontaneous activity, we induced desynchronized activity. The transformation of the activity is illustrated in the raw recordings (Fig. 5*A*) and in the spectrogram (Fig. 5*B*). We then bath-applied increasing concentrations of CGP55845, a specific antagonist of GABA_B_-Rs. Such application had a progressive effect enhancing the synchronization in the network (Fig. 5*A*,*B*) although it did not turn activity into epileptiform activity as GABA_A_-R blockade did (Fig. 3*A*,*B*). We computed the sPCI for each condition (Fig. 5*C–E*) and significant differences were found (*F*_(5,30)_ = 2.921, *p* = 0.029; *n* = 7). Although sPCI significantly increased during desynchronized condition compared with SO (*t*_(30)_ = 4.75, *p* = 0.024, *n* = 7; Fig. 5*E*), *post hoc* tests did not reveal significant differences, neither between control versus CGP conditions (CGP 100 nm: *t*_(30)_ = 2.87, *p* = 0.348; CGP 200 nm: *t*_(30)_ = 1.68, *p* = 0.837; CGP 500 nm: *t*_(30)_ = 1.29, *p* = 0.941; CGP 1 μm: *t*_(30)_ = 0.69, *p* = 0.996; *n* = 7; Fig. 5*E*) nor desynchronized versus CGP groups (CGP 100 nm: *t*_(30)_ = 1.88, *p* = 0.768; CGP 200 nm: *t*_(30)_ = 3.07, *p* = 0.281; CGP 500 nm: *t*_(30)_ = 3.47, *p* = 0.171; CGP 1 μm: *t*_(30)_ = 4.07, *p* = 0.072; *n* = 7; Fig. 5*E*). The temporal evolution of sPCI (Fig. 5*D*) also revealed an increase in the desynchronized condition, but a similar evolution in SO and under GABA_B_-R blockade, conditions with an increased synchronization and thus lesser spatiotemporal richness in the patterns. In summary, these results indicate that blockade of GABA_B_-Rs during desynchronized activity showed a trend toward a decreased sPCI but did not reach significance. Thus, the contribution of GABA_B_-mediated inhibition to causal complexity in the awake state is less relevant than that of GABA_A_-mediated inhibition.

### Role of GABA_B_-Rs in the modulation of cortical dynamics and complexity: blocking of GABA_B_-Rs in the slow oscillatory state

**Figure 6.**
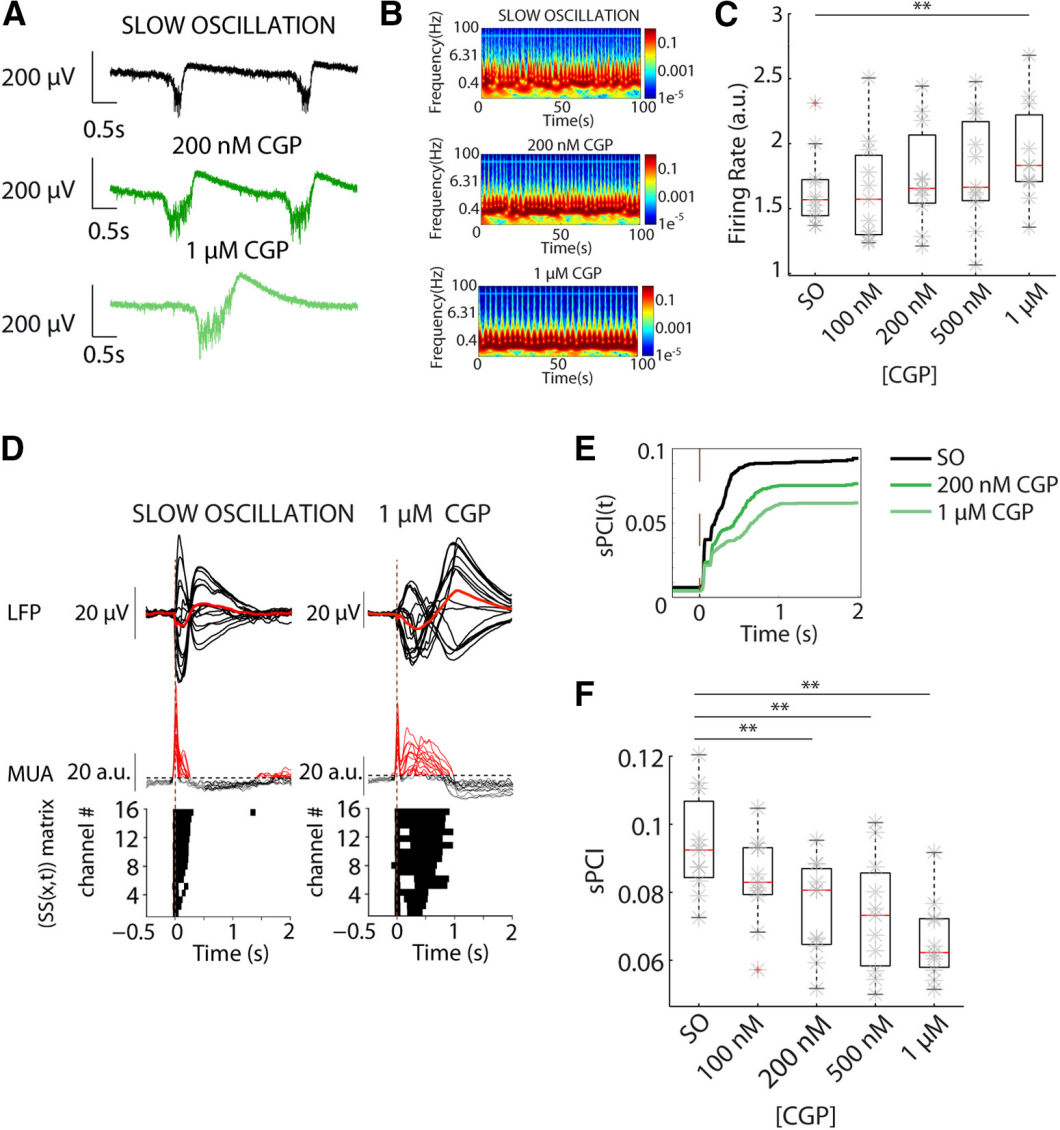
Progressive blockade of GABA_B_-Rs reduces sPCI during SO leading stereotypical activity. ***A***, Raw LFP recordings of spontaneous activity in neocortical slices, control SOs and blocking GABA_B_-Rs by bath application of increasing concentrations of CGP, which induced Up states of longer duration followed by prominent Down states, decreasing the Up-state frequency and increasing the regularity. ***B***, Spectrograms of spontaneous activity from LFP recordings shown in ***A***. ***C***, Population of relative FRs of increasing concentrations of CGP. ***D***, Averaged LFP (top) and MUA (middle) to electrical stimulation during distinct regimes of activity. Binary matrices of significant sources of activity [SS(*x*,*t*)] following electrical stimulation delivered to neocortical slices (bottom). ***E***, Time evolution of sPCI in the three experimental conditions. ***F***, Population sPCI (*n* = 11; **p < 0.01).

During SO, GABA_B_-Rs have been found to play a role in Up-state termination since their blockade results in longer persistent activity (Mann et al., 2009; Perez-Zabalza et al., 2020; Sanchez-Vives et al., 2021). In order to further investigate the role of GABA_B_-R-mediated inhibition in emergent activity and cortical complexity, we gradually blocked GABA_B_-Rs while departing from slow oscillatory activity. Progressive blockade of GABA_B_-Rs induced Up states of longer duration followed by prominent Down states that decreased the frequency of Up states and increased their regularity, as described in (Perez-Zabalza et al., 2020; Fig. 6*A*,*B*). The spectrograms illustrate the enhanced synchronization (Fig. 6*B*). The sPCI decreased following GABA_B_-R blockade (*F*_(4,40)_ = 9.351, *p* = 1.97 × 10^−5^; *n* = 11; Fig. 6*D–F*). The time evolution of sPCI (Fig. 6*E*) also decreased with GABA_B_-R blockade. Significant sPCI reductions were confirmed by *post hoc* analysis for the three tested conditions (CGP 200 nm: *t*_(40)_ = 5.38, *p* = 0.004; CGP 500 nm: *t*_(40)_ = 5.87, *p* = 0.002; CGP 1 μm: *t*_(40)_ = 7.98, *p* = 1.45 × 10^−5^; *n* = 11). Interestingly, the removal of slow ′inhibition by bath-application of increasing concentrations of CGP55845 significantly increased the firing rate during Up states (*F*_(1.84,18.42)_ = 4.481, *p* = 0.028; *n* = 11; Fig. 6*C*), although to a lesser extent than GABA_A_-R blockade did. Only bath-application of 1 μm CGP55845 resulted in a significant increase of firing rate (*t*_(40)_ = 4.996, *p* = 0.009, *n* = 11). Finally, we showed that blockade of GABA_B_-Rs, while in SO, reduced perturbational complexity, confirming that GABA_B_-R-mediated inhibition contributes to the richness of activity patterns, spatiotemporal variability, and cortical complexity during the slow oscillatory regime.

### The role of cortical GABA_A_-Rs and GABA_B_-Rs in the modulation of cortical complexity in a cortical network model

**Figure 7.**
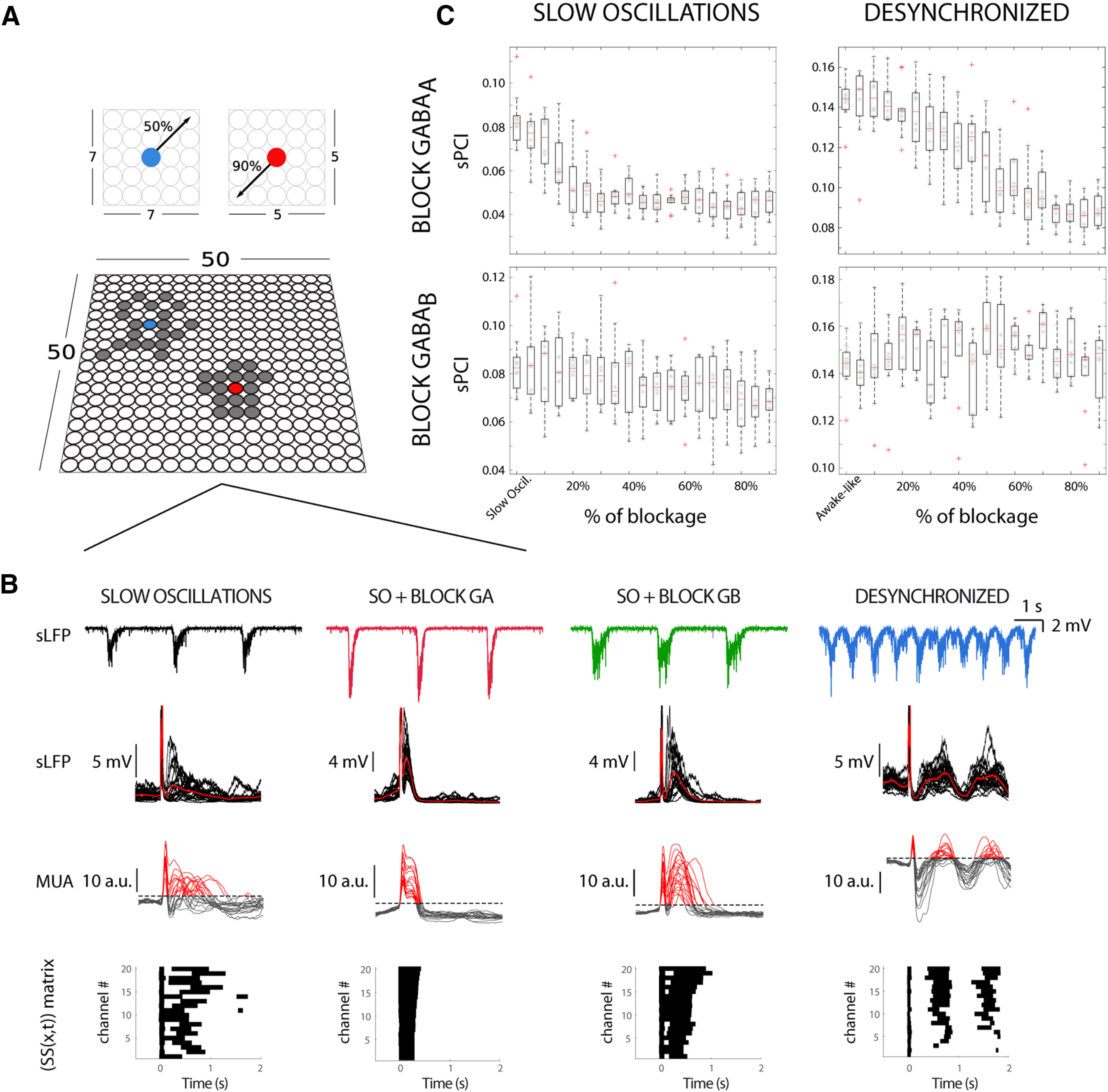
A network cortical model to reproduce the sPCI measured during different regimes of network activity. ***A***, The model consists of pyramidal (blue) and inhibitory neurons (red) arranged in a 50 × 50 square lattice. The excitatory neurons may connect locally to a 50% fraction of its neighbors (gray circles) within a 7 × 7 square, while the inhibitory neurons to a 90% fraction within a 5 × 5 square (see Materials and Methods). All details of the model are in Extended Data Figure 7-1. ***B***, The model reproduces similar spontaneous and evoked neuronal activity, as observed experimentally during (from left to right) SOs, SOs + blocking GABA_A_ (SO + BLOCK G_A_), SOs + blocking GABA_B_ (SO + BLOCK G_B_) and desynchronized activity. Single spontaneous sLFP (top), averaged sLFP (middle top), MUA (middle bottom), and binary matrices of significant sources of activity [SS(*x,t*)] following stimulation delivered in a cortical model. ***C***, Population sPCI for different cortical activity, SOs with GABA_A_ (left-top) and GABA_B_ (left-bottom) progressively blockade, the same for desynchronized activity (right column).

In order to further investigate the cellular and network mechanisms involved in the spatiotemporal dynamics of spontaneous and induced cortical complexity, we implemented a modified version of a biophysically detailed neuronal model (Compte et al., 2003) in a two-dimensional network. The model consists of pyramidal and inhibitory conductance-based neurons synaptically connected within a local range. Pyramidal cells have a larger range of connectivity than inhibitory neurons, which are more locally connected (Fig. 7*A*; for details, see Materials and Methods). Our neuronal model includes GABA_A_ as in Compte et al. (2003) and additionally accounts for GABA_B_ inhibitory synapses, as well as potassium leakage current which is modulated by ACh and NE (Bazhenov et al., 2002; Li et al., 2017). Equations and parameters of the model can be found in Extended Data Fig. 7-1. In this model, we simulated population LFPs (sLFP), and recorded from 20 different locations organized in a matrix (see Materials and Methods).

**Figure 8.**
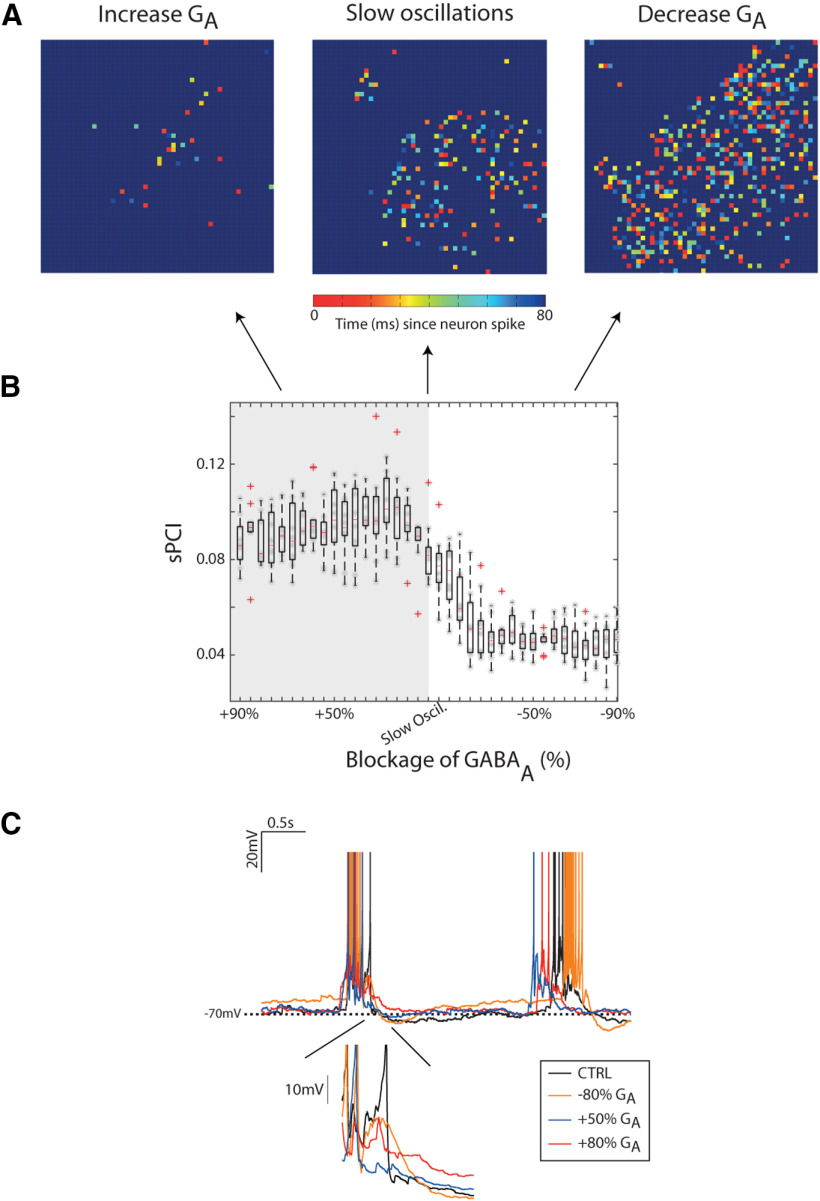
sPCI for an inhibited and disinhibited cortical network model. ***A***, Example snapshot of cortical network activity for three networks with different inhibition (GABA_A_) concentration (E/I balance). The color scale indicates the time since each neuron last spiked, thus illustrating the temporal dynamics of activity propagation. Right, Networks with low inhibition blockade (−80%) show high integration, while with high inhibition blockade (+80%) show high segregation (left). Middle, Networks with an intermediate segregation/integration balance. See the simulations in Movie 1. ***B***, Population sPCI for inhibited and disinhibited cortical networks. The shadow area represents the model predictions. ***C***, Membrane potential traces for three neurons in the cortical network model during SOs (CTRL), disinhibited (orange), slightly inhibited (red), and high inhibited (blue) cortical network activity. Notice the increase and decrease afterhyperpolarization (AHP) following the up state for slightly inhibited and high inhibited neurons, respectively.

The sLFP signal was analyzed with exactly the same techniques as the ones experimentally recorded in the cortical slices. The model is able to reproduce slow oscillatory dynamics and desynchronized activity as observed *in vitro*, as well as the cortical activity under blockade of GABA_A_-Rs and GABA_B_-Rs (Fig. 7*B*; see Movie 1). The transition from synchronized toward desynchronized activity was modeled by blocking the potassium leak current, mimicking the action of ACh and NE (McCormick, 1992), a strategy that has been used in thalamocortical models to this end (Bazhenov et al., 2002; Li et al., 2017; see Materials and Methods). We then evaluated the perturbational complexity in the cortical network model. For SO and desynchronized activity, the sPCI showed similar values to those observed *in vitro*: 0.08 ± 0.01 and 0.14 ± 0.01, respectively. We next tested the effects of the progressive blockade of GABA_A_ and GABA_B_ during both different dynamics corresponding to the conditions of SO and desynchronized states. The maximal effect of GABA_A_ blockade on the sPCI during SO occurred by reducing the receptor availability by 20%, when we obtained values of sPCI 0.05 ± 0.01 that remained unchanged for lower availability (Fig. 7*C*, top left). On the other hand, the sPCI during desynchronized dynamics progressively decreased with the GABA_A_-R blockade, reaching a plateau for blockade of >80% of receptors, 0.08 ± 0.01 (Fig. 7*C*, top right). Interestingly, for large GABA_A_ blockade during desynchronized activity, the sPCI values approach those observed during SO, as observed experimentally. For the GABA_B_ -Rs blockade, we observed a progressive slow decay of the sPCI values from SO conditions (Fig. 7*C*, bottom left), while for desynchronized dynamics we did not observe any trend in sPCI (Fig. 7*C*, bottom right). To a lesser extent, the GABA_B_ effects were also similar to those observed experimentally. Since GABA_A_ modulation presented a stronger effect on the perturbational complexity in both conditions (i.e., SO and desynchronized dynamics), we next proceeded to evaluate the network dynamics in two scenarios: (1) in a disinhibited network and (2) in an inhibited network. As observed experimentally during SO, when we blocked GABA_A_ (i.e., disinhibited the network) the spontaneous activity presented a shorter Up state with higher firing rate (Fig. 7*B*, compare top left and top middle left). In the model, we observed that the dynamics of spontaneous activity in a disinhibited network during SO is fully integrated, while weakly segregated, giving rise to activation waves that rapidly span the whole network (Fig. 8*A*, right; see Movie 1). Conversely, when the network is inhibited, the spontaneous activity is highly segregated and weakly integrated, and the activation waves propagate more locally and do not span over the whole network (Fig. 8*A*, left; Movie 1). Nonetheless, when there is a balance between integration and segregation, the activation waves span over the whole network recruiting their nearest neighbors (Fig. 8*A*, middle; Movie 1). Finally, we evaluated the perturbational complexity networks where the inhibition was not only decreased (as in the experiments), but also increased. While departing from the slow oscillatory regime, we found that increasing inhibition by +20% further increased sPCI (0.10 ± 0.01), remaining high during SO for highly inhibited networks (+90%, 0.09 ± 0.02; Fig. 8*B*). The increment on the sPCI values for slightly more inhibited networks may be because of the fact that the neurons on the network fired less during the Up states, and therefore had less hyperpolarization at the beginning of Down states (Fig. 8*C*), shortening the Down states and allowing a less synchronized and bistable network. These results suggest that there is a close link between integration and segregation with E/I balance, and that higher/lower sPCI values are not the consequence of merely increasing/decreasing excitability.

Movie 1. Spatiotemporal dynamics of the SO in a cortical network model under different regimes of inhibition. The movie shows three different dynamical states as a function of inhibition: GABA_A_ is increased by 80% (inhibited state, left), control condition (balanced state, center) and GABA_A_ is decreased by 80% (disinhibited state, right). L indicates the size of the squared network (here L = 50). The color code indicates the time since the last neuronal spike (for details, see Materials and Methods). 10.1523/JNEUROSCI.1837-20.2021.video.1

## Discussion

In this study we have investigated the role of GABA_A_-R-mediated and GABA_B_-R-mediated inhibition on cortical emergent activity and complexity, in particular on complexity measured by means of perturbing the network with stimulation. By doing this we have attempted to bridge a macroscale clinical measure (PCI), with the synaptic and cellular components of the local cortical circuits. We found that during physiological activity, both types of inhibition, fast and slow, contribute to the generation of richness of spatiotemporal activity patterns and cortical complexity, and the progressive blockade of fast or slow inhibition results in enhanced synchronization and breakdown of complexity. However, the contribution of GABA_A_-R-mediated and GABA_B_-R-mediated inhibition is different in the desynchronized and in the slow oscillatory regimes, and this is discussed below. In our computational model, we explore areas of the parameter space that cannot be reached experimentally, exploring more extensively the relationship between excitatory and inhibitory balance, network dynamics, and cortical complexity.

The PCI (Casali et al., 2013; Comolatti et al., 2019) is a measure of cortical complexity that has been used to quantify consciousness levels, in awake/sleep, in anesthesia (Hudetz, 2012; Sarasso et al., 2015) and in patients with disorders of consciousness (Rosanova et al., 2012; Casarotto et al., 2016). This measure consists in the perturbation of the brain network by means of cortical stimulation to engage the corticothalamocortical circuit in causal interactions, and then capturing the spatiotemporal properties of the response in an index that reflects cortical complexity. It is based on the hypothesis that for consciousness to occur, simultaneous integration and segregation of information in the network are needed, resulting in high complexity in the awake state (Tononi and Edelman, 1998). Network complexity can be measured by different means in the spontaneous activity, either electrophysiological or imaging signal. A variety of such measures exist, including Lempel–Ziv compressibility (Szczepański et al., 2003; Hudetz et al., 2016), Shannon entropy (Zhao et al., 2010), entropy of wave propagation (Barbero-Castillo et al., 2019), and functional complexity (Zamora-López et al., 2016), among others. However, a perturbational approach presents advantages with respect to an observational one (based on spontaneous activity) because it is less affected by noise or isolated processes, and only assesses information generated through deterministic interactions, which also gives advantages that are useful clinically (Casali et al., 2013). Highly synchronized states (slow wave sleep or anesthesia) display bistable responses to stimulation, which prevents the cortical network engaging in a chain of complex causal interactions that are typical of the awake state (Massimini et al., 2005, 2007; Pigorini et al., 2015). In such “bistable” states, the network falls into an off-period following stimulation (Rosanova et al., 2018). While highly synchronized states like slow wave sleep or deep anesthesia show low complexity and the awake state, high complexity, in between there are intermediate brain states and intermediate complexity levels, which can be investigated using various anesthetics (Sarasso et al., 2015), at different levels of anesthesia (Dasilva et al., 2021) or in different disorders of consciousness (Casarotto et al., 2016). Given that there is accumulating evidence that brain complexity is a relevant property that informs about the brain state and consciousness levels, we want to link this measure to the properties of cells and circuits, to understand how the different mechanisms may sculpt the resulting complexity. This understanding is also important to eventually devise strategies to recover complexity in pathologic situations.

In order to investigate how inhibition contributes to complexity, we resorted to the progressive blockade of GABAergic receptors. However, network complexity in humans has been found to be different in the synchronized, slow oscillatory state (low complexity) and in the awake state (high complexity; see above). For this reason, we conducted the GABAergic blockade while departing from these two extreme conditions, to understand the different role GABAergic inhibition plays in both regimes. SO dynamics is a multiscale phenomenon which emerges in cortical circuits whenever there is physical or functional disconnection of the cortex, such as NREM sleep or anesthesia (Steriade et al., 1993a; Sanchez-Vives and McCormick, 2000; Massimini et al., 2004; Riedner et al., 2007; Alkire et al., 2008; Chauvette et al., 2011). For this reason, SO has been suggested to represent the “default” pattern of activity of the cortical network (Sanchez-Vives and Mattia, 2014), which emerges even in isolated cortical tissue *in vitro* (Sanchez-Vives and McCormick, 2000). From synchronized regimes of SO, neural activity can transition toward desynchronized states leading to variable, irregular and spatiotemporally complex cortical rhythms, in which neurons fire irregularly and nearly independently during the awake state (Steriade et al., 2001; Chen et al., 2009; Constantinople and Bruno, 2011; Duarte et al., 2017; Andalman et al., 2019; Poulet and Crochet, 2019). The cellular substrate underlying the switch between SO and desynchronized activity is provided, among others, by ascending cholinergic and noradrenergic neuromodulatory connections arising from subcortical structures, such as the basal forebrain or locus coeruleus (Hobson and Pace-Schott, 2002; Jones, 2005; Weber and Dan, 2016; Scammell et al., 2017). Our preparation reproduced several features of these different dynamics. Bath-application of NE+CCh shifted SO to low-frequency 1- to 5-Hz oscillations (Fig. 2*A–C*; as in D'Andola et al., 2017), which in part resemble the cortical activity observed during wakefulness. Further, we lowered the temperature by 2°C to 32°C, which we have demonstrated previously diminishes cortical synchronization (Reig et al., 2010), and increases excitability by increasing electrical compactness and synaptic summation (Trevelyan and Jack, 2002). Further, we lowered [Ca^2+^]_o_ levels to enhance excitability (0.8–0.9 mm; Markram et al., 2015). Both manipulations resulted in larger desynchronization as illustrated in the autocorrelograms (Fig. 2*B*) and spectrograms (Fig. 2*C*). Both manipulations led to higher sPCI values (Fig. 2*D*) as well as increased SampEn with respect to the SO regime, which is consistent with the idea of different cortical dynamics. Our experimental model thus allows the study of transitions between different cortical dynamics, SO to desynchronized states.

Departing from SO as well as from desynchronized activity, GABA_A_-R blockade resulted in Up states of higher amplitude and shorter duration than those observed in control conditions (Fig. 4). This property has been described (Sanchez-Vives et al., 2010), but only for the evolution of Up states in SO. Interestingly, when departing from desynchronized activity, it is also possible to induce rhythmicity in low frequencies by partially blocking GABA_A_-Rs. It should be noticed that this is not epileptiform activity, although progressive inhibition would eventually lead to epileptiform discharges. Disinhibition causes higher firing rates that induces a hypersynchronization of the network, as we also find in our computer model simulations. In the simulations, the activation of potassium channels is critical to induction of the silent periods. Such hypersynchronization of the network results in a decrease in complexity.

GABA_B_-R blockade during SOs increased Up-state duration (Mann et al., 2009; Perez-Zabalza et al., 2020), resulting in highly regular oscillatory patterns and prominent bistable responses to electrical perturbation (Fig. 6). This finding is revealing of the role of GABA_B_-Rs in increasing richness of activity patterns and irregularity in the Up and Down states (Perez-Zabalza et al., 2020). This is also translated in a consistently decreased sPCI with GABA_B_-R blockade. A different situation takes place when departing from the desynchronized state. In this situation, the blockade of GABA_B_-Rs tends to induce, but does not fully induce, a bistable oscillatory regime, nor significantly decreases sPCI. These findings suggest that the role of GABA_B_-Rs in the desynchronized state for introducing richness of activity patterns and thus complexity, is not as relevant as it is for the SO regime. It is probably the case that the intense firing of neurogliaform neurons necessary for the activation of GABA_B_-Rs is more commonly achieved in synchronized than in desynchronized states (for review, see Craig and McBain, 2014; Sanchez-Vives et al., 2021).

### Local network excitability, bistability, and the integration–segregation balance in brain slices

A straightforward explanation of our observation of increased sPCI during desynchronized states is that the sPCI merely reflects network excitability. In other words, the sPCI scored higher just because bath-application of modulators such as CCh or NE increased the excitability of the slice. However, if that were the case, any experimental manipulation increasing the excitability of the slice would result in higher sPCI values compared with those obtained under the SO regime (Barbero-Castillo et al., 2019; D'Andola et al., 2017) already demonstrated the opposite, showing that either bath-application of a glutamate receptor agonist (kainate) or electric field modulation (respectively) in cortical slices increased network excitability without affecting sPCI. Finally, the authors did not find a relationship between network excitability and sPCI (D'Andola et al., 2017). Here, we further explored the relationship between excitability, inhibition and connectivity in our experiments and computer model and provided a number of novel insights. On one hand, the progressive reduction of fast inhibition gradually increased the firing rate as illustrated in the figures, even leading to epileptiform discharges in some cases. We found that excessive excitability can in fact reduce the sPCI, and statistical analysis indicated that following GABA_A_-R blockade, the sPCI was significantly reduced while the relative firing rate increased. On the other hand, GABA_B_-R blockade also reduced the sPCI without significantly increasing the firing rate, supporting the idea of the independence of firing rates or excitability and complexity.

Finally, our computer model allowed us to investigate areas of the parameter space that were not visited experimentally. The model that has been used for these simulations, as described, is a biologically inspired, Hodgkin and Huxley model (Hodgkin and Huxley, 1952), in which pyramidal neurons have two compartments: somatic and dendritic (see Materials and Methods), where excitatory synapses target the dendritic compartment and inhibitory synapses are localized on the somatic compartment of postsynaptic pyramidal neurons (Compte et al., 2003). This network model was originally tuned based on experimental observations of cortical SOs, and further studies have validated that it reproduces diverse features of excitability and oscillations (Sancristóbal et al., 2016), and of cortical emergent properties such as β and γ frequencies (Compte et al., 2008). Activation of GABAergic receptors in the soma compartment hyperpolarizes the membrane potential and decreases the input resistance, while those in dendrites modulate NMDA potentials (Doron et al., 2017), calcium spikes, block back-propagation of action potentials and neuronal bursting, all without modifying the membrane potential in the soma (Larkum et al., 1999; Pouille and Scanziani, 2004; Breton and Stuart, 2012; Palmer et al., 2012; Pouille et al., 2013). In a previous study, we demonstrated that this model could reproduce at the cellular and network level the transformation of the SOs when GABA_A_-Rs were progressive blocked (Sanchez-Vives et al., 2010). In the current study, we aimed to reproduce in our model population features that we observed while blocking as well GABA_B_-R: an elongation of the Up states (as in Mann et al., 2009; Perez-Zabalza et al., 2020), and the spatiotemporal response to stimulation (or perturbation), which results in a decrease in the resulting PCI with GABAergic block. Somatic GABAergic receptors located in the soma in our model were sufficient to reproduce both spontaneous and evoked population responses observed in cortical slices, although a further exploration of a more detailed somatic versus dendritic influence on network dynamics could be a valuable future development. In the model we explored parametrically the variation of GABA_A_-R blockade and found the rate of decrease of sPCI reached a minimum for about a 25% decrease in inhibition. Interestingly, we were also able to enhance inhibition, and found that there is a window of excitatory/inhibitory balance, around the physiological values, in which complexity is maximal, but either enhancing or decreasing inhibitions diminishes complexity. However, when we look at the spatial patterns (see Movie 1) we can see how the spatiotemporal pattern to reach decreased complexity can be very different, from a highly disaggregated activity in enhanced inhibition, to hypersynchronization in low inhibition. This finding bridges the activity of receptors with the activation of the network at the mesocortical level and connects circuit properties with large-scale causal interactions in the cortical network.
