# Supplementary material for: Impact of GABAA and GABAB Inhibition on Cortical Dynamics and Perturbational Complexity during Synchronous and Desynchronized States
Source: J Neurosci. 2021 Jun 9;41(23):5029–44. doi: 10.1523/JNEUROSCI.1837-20.2021 (PMC8197642; doi:10.1523/JNEUROSCI.1837-20.2021)
Supplement: Figure 7-1 — Computational network model. Download Figure 7-1, DOCX file. [file ns-JN-RM-1837-20-s02.docx]

**Figure 7-1. Computational network model.**

| *Description* | *Equations* | *Parameters* | *Reference* |
| --- | --- | --- | --- |
| Hodgkin-Huxley formalism | $\frac{dx}{dt}= Ф\left[ \alpha_{x}\left( V \right)\left( 1-x \right){-\beta}_{x}\left( V \right)x \right]$  $\frac{dx}{dt}=\frac{Ф}{\tau_{x}(V)}[x_{\infty}\left( V \right)-x]$ | $Ф=1$  (unless otherwise stated) | (Hodgkin and Huxley, 1952) |
| Sodium current (PY-Soma) | $I_{Na}=g_{Na}m_{\infty}^{3}{h(V-V}_{Na})$  $m_{\infty}=\alpha_{m}(\alpha_{m}+\beta_{m})$  $\alpha_{m}=\frac{0.1\left( V+33 \right)}{\left[ 1-\exp\left( -0.1\left( V+33 \right) \right) \right]}$  $\beta_{m}=4 e\mathrm{xp}(-\frac{1}{12}(V+53.7))$  $\alpha_{h}=0.07 exp(-0.1(V+50))$  $\beta_{h}=\frac{1}{\left[ 1+\exp\left( -0.1\left( V+20 \right) \right) \right]}$ | $g_{Na}=50\frac{mS}{cm^{2}}$  $V_{Na}=55 mV$  $Ф=4$ | (Compte et al., 2003) |
| Potassium current  (PY-Soma) | $I_{K}=g_{K}n^{4}{(V-V}_{K})$  $\alpha_{n}=\frac{0.01\left( V+34 \right)}{\left[ 1-\exp\left( -0.1\left( V+34 \right) \right) \right]}$  $\beta_{n}=0.125 e\mathrm{xp}(-\frac{1}{25}(V+44))$ | $g_{K}=10.5\frac{mS}{cm^{2}}$  $V_{K}=-100 mV$  $Ф=4$ | (Compte et al., 2003) |
| Leakage current  (PY-Soma&Dendrite) | $I_{L}=g_{L}{(V-V}_{L})$ | $V_{L}=-60.95\pm0.3 mV$  $g_{L}=0.0667$  $\pm0.0067\frac{mS}{cm^{2}}$  $(mean\pm std)$ | (Compte et al., 2003) |
| Potassium leakage current  (PY-Soma) | $I_{KL}=g_{KL}{(V-V}_{K})$ | $V_{K}=-100 mV$  $g_{KL}=1.86\frac{mS}{cm^{2}}$ | (Li et al., 2017) |
| Fast A-type K^+^ current  (PY-Soma) | $I_{A}=g_{A}m_{\infty}^{3}{h(V-V}_{K})$  $m_{\infty}=\frac{1}{\left[ 1+\exp\left( -0.05\left( V+50 \right) \right) \right]}$  $h_{\infty}=\frac{1}{\left[ 1+\exp\left( 0.16\left( V+80 \right) \right) \right]}$ | $V_{K}=-100 mV$  $g_{A}=1\frac{mS}{cm^{2}}$  $\tau_{h}=15 ms$ | (Compte et al., 2003) |
| Non-inactivating K^+^ current  (PY-Soma) | $I_{KS}=g_{KS}{m(V-V}_{K})$  $m_{\infty}=\frac{1}{\left[ 1+\exp\left( -\frac{1}{6.5}\left( V+34 \right) \right) \right]}$  $\tau_{m}=\frac{8}{\left[ \exp\left( -\frac{1}{30}\left( V+55 \right) \right)+\exp\left( \frac{1}{30}\left( V+55 \right) \right) \right]}$ | $V_{K}=-100 mV$  $g_{KS}=0.576\frac{mS}{cm^{2}}$ | (Compte et al., 2003) |
| Na^2+^-dependent K^+^ current  (PY-Soma) | $\frac{d[{Na}^{+}]}{dt}=-\alpha_{Na}\left( A_{s}I_{Na}+A_{d}I_{NaP} \right)-R_{pump}\{\frac{\left[ Na^{+} \right]^{3}}{\left[ Na^{+} \right]^{3}+{15}^{3}}-\left[ Na^{+} \right]_{eq}^{3}/(\left[ Na^{+} \right]_{eq}^{3}+{15}^{3})\}$  $I_{KNa}=g_{KNa}w_{\infty}([{Na}^{+}])(V-V_{K})$  $w_{\infty}{([Na}^{+}])=0.37/\left[ 1+{(38.7/{[Na}^{+}])}^{3.5} \right]$ | $V_{K}=-100 mV$  $\alpha_{Na}=0.01\frac{mM}{nA\cdot ms}$  $R_{pump}=0.018\frac{mM}{ms}$  ${[Na^{+}]}_{eq}=9.5 mM$  $g_{KNa}=0.65835\frac{mS}{cm^{2}}$ | (Compte et al., 2003) |
| Persistent sodium current  (PY-Dendrite) | $I_{NaP}=g_{NaP}{m_{\infty}^{3}(V-V}_{Na})$  $m_{\infty}=\frac{1}{\left[ 1+\exp\left( -\frac{1}{7.7}\left( V+55.7 \right) \right) \right]}$ | $V_{Na}=55 mV$  $g_{NaP}=0.05145\frac{mS}{cm^{2}}$ | (Compte et al., 2003) |
| Rectifier K^+^ current  (PY-Dendrite) | $I_{AR}=g_{AR}{h_{\infty}(V-V}_{K})$  $h_{\infty}=\frac{1}{\left[ 1+\exp\left( 0.25\left( V+75 \right) \right) \right]}$ | $V_{K}=-100 mV$  $g_{AR}=0.0257\frac{mS}{cm^{2}}$ | (Compte et al., 2003) |
| High-threshold Ca^2+^ current  (PY-dendrite) | $I_{Ca}=g_{Ca}{m_{\infty}^{2}(V-V}_{Ca})$  $m_{\infty}=\frac{1}{\left[ 1+\exp\left( -\frac{1}{9}\left( V+20 \right) \right) \right]}$ | $V_{Ca}=120 mV$  $g_{Ca}=0.43\frac{mS}{cm^{2}}$ | (Compte et al., 2003) |
| Ca^2+^-dependent K^+^ current  (PY-Dendrite) | $\frac{d[{Ca}^{2+}]}{dt}=-\alpha_{Ca}A_{d}I_{Ca}-\frac{\left[ {Ca}^{2+} \right]}{\tau_{Ca}}$  $I_{KCa}=\frac{g_{KCa}\left[ {Ca}^{2+} \right]}{\left[ {Ca}^{2+} \right]+K_{D}}{(V-V}_{K})$ | $\alpha_{Ca}=0.005\frac{\mu M}{nA\cdot ms}$  $\tau_{Ca}=150 ms$  $K_{D}=30 \mu M$  $V_{K}=-100 mV$  $g_{KCa}=0.5415\frac{mS}{cm^{2}}$ | (Compte et al., 2003) |
| Sodium current (IN-Soma) | $I_{Na}=g_{Na}m_{\infty}^{3}{h(V-V}_{Na})$  $m_{\infty}=\alpha_{m}(\alpha_{m}+\beta_{m})$  $\alpha_{m}=\frac{0.5\left( V+35 \right)}{\left[ 1-\exp\left( -0.1\left( V+35 \right) \right) \right]}$  $\beta_{m}=20 e\mathrm{xp}(-\frac{1}{18}(V+60))$  $\alpha_{h}=0.35 exp(-\frac{1}{20}(V+58))$  $\beta_{h}=\frac{5}{\left[ 1+\exp\left( -0.1\left( V+28 \right) \right) \right]}$ | $g_{Na}=35\frac{mS}{cm^{2}}$  $V_{Na}=55 mV$ | (Compte et al., 2003) |
| Potassium current  (IN-Soma) | $I_{K}=g_{K}n^{4}{(V-V}_{K})$  $\alpha_{n}=\frac{0.05\left( V+34 \right)}{\left[ 1-\exp\left( -0.1\left( V+34 \right) \right) \right]}$  $\beta_{n}=0.625 e\mathrm{xp}(-\frac{1}{80}(V+44))$ | $g_{K}=9\frac{mS}{cm^{2}}$  $V_{K}=-90 mV$ | (Compte et al., 2003) |
| Leakage current  (IN-Soma) | $I_{L}=g_{L}{(V-V}_{L})$ | $V_{L}=-63.8\pm0.15 mV$  $g_{L}=0.1025$  $\pm0.0025\frac{mS}{cm^{2}}$  $(mean\pm std)$ | (Compte et al., 2003) |
| PY/IN-Neuron parameters | - | $C_{m}=1\frac{\mu F}{cm^{2}}$  $A_{s}=0.015mm^{2}$  $A_{d}=0.035mm^{2}$  $A_{i}=0.02mm^{2}$  $g_{sd}=1.75\pm0.1\mu S$ | (Compte et al., 2003) |
| GABA-A synaptic current | $I_{GABA-A}=g^{XY}s(t)(V-V_{syn})$  $\frac{ds}{dt}=\alpha{f(V}_{pre})-\frac{s}{\tau}$  ${f(V}_{pre})=1/\left[ 1+exp(-0.5(V_{pre}-20)) \right]$ | $g^{II}=1.96 nS$  $g^{IE}=28.96 nS$  $\alpha=1$  $\tau=10 ms$  $V_{syn}=-70 mV$ | (Compte et al., 2003) |
| GABA-B synaptic current | $I_{GABA-B}=g^{XY}\frac{s^{4}}{s^{4}+K_{g}}(V-V_{syn})$  $\frac{dr}{dt}=0.5T\left( 1-r \right)-0.0012r$  $\frac{ds}{dt}=0.18r-0.034s$ | $g^{IE}=39.2 nS$  $g^{II}=33.75 nS$  T is modeled as a square pulse of $0.5 mM$ during$3 ms$.  $K_{g}=100 \mu M^{4}$  $V_{syn}=-85 mV$ | (Destexhe et al., 1996; Liu et al., 2019) |
| AMPA synaptic current | $I_{AMPA}=g^{XY}s(t)(V-V_{syn})$  $\frac{ds}{dt}=\alpha{f(V}_{pre})-\frac{s}{\tau}$  ${f(V}_{pre})=1/\left[ 1+exp(-0.5(V_{pre}-20)) \right]$ | $g^{EI}=0.6925 nS$  $g^{EE}=6.2 nS$  $\alpha=3.48$  $\tau=2 ms$  $V_{syn}=0 mV$ | (Compte et al., 2003) |
| NMDA synaptic current | $I_{NMDA}=\frac{g^{XY}s\left( t \right)\left( V-V_{syn} \right)}{1+{[Mg}^{2+}]exp(-\frac{0.062V}{3.57})}$  $\frac{ds}{dt}=\alpha z\left( 1-s \right)-\frac{s}{\tau}$  $\frac{dz}{dt}=\alpha_{z}f\left( V_{pre} \right)-\frac{z}{\tau_{z}}$ | $g^{EI}=0.595 nS$  $g^{EE}=2.72 nS$  $\alpha=0.5$  $\tau=100 ms$  $\alpha_{z}=3.48$  $\tau_{z}=2 ms$  $V_{syn}=0 mV$ | (Compte et al., 2003) |
| Poisson noise | The Poisson synapses are modeled as excitatory AMPA and NMDA currents where the probability of a spike at one time step is given by:$1-exp(-R*dt)$, where $dt$ is the time step of simulation, and R the Poisson rate. | $g_{AMPA}^{EI}=0.0004625 nS$  $g_{AMPA}^{EE}=0.0012 nS$  $g_{NMDA}^{EI}=0.00095 nS$  $g_{NMDA}^{EE}=0.0004 nS$  $R=0.5 kHz$ | (Dayan and Abbott, 2001) |

PY= Pyramidal neuron; IN= Inhibitory neuron; g^XY^ (maximal conductance from X to Y)
